# Can particulate matter be identified as the primary cause of the rapid spread of CoViD-19 in some areas of Northern Italy?

**DOI:** 10.1007/s11356-021-12735-x

**Published:** 2021-02-26

**Authors:** Maria Cristina Collivignarelli, Alessandro Abbà, Francesca Maria Caccamo, Giorgio Bertanza, Roberta Pedrazzani, Marco Baldi, Paola Ricciardi, Marco Carnevale Miino

**Affiliations:** 1 Department of Civil Engineering and Architecture, University of Pavia, via Ferrata 3, 27100 Pavia, Italy; 2 Interdepartmental Centre for Water Research, University of Pavia, via Ferrata 3, 27100 Pavia, Italy; 3 Department of Civil, Environmental, Architectural Engineering and Mathematics, University of Brescia, via Branze 43, 25123 Brescia, Italy; 4 Department of Mechanical and Industrial Engineering, University of Brescia, via Branze 38, 25123 Brescia, Italy; 5 Department of Chemistry, University of Pavia, viale Taramelli 10, 27100 Pavia, Italy

**Keywords:** PM_10_, PM_2.5_, SARS-CoV-2, Doubling time, Coronavirus, Epidemic

## Abstract

Northern Italy was the most affected by CoViD-19 compared to other Italian areas and comprises zones where air pollutants concentration was higher than in the rest of Italy. The aim of the research is to determine if particulate matter (PM) has been the primary cause of the high CoViD-19 spread rapidity in some areas of Northern Italy. Data of PM for all the 41 studied cities were collected from the local environmental protection agencies. To compare air quality data with epidemiological data, a statistical analysis was conducted identifying the correlation matrices of Pearson and Spearman, considering also the possible incubation period of the disease. Moreover, a model for the evaluation of the epidemic risk, already proposed in literature, was used to evaluate a possible influence of PM on CoViD-19 spread rapidity. The results exclude that PM alone was the primary cause of the high CoVid-19 spread rapidity in some areas of Northern Italy. Further developments are necessary for a better comprehension of the influence of atmospheric pollution parameters on the rapidity of spread of the virus SARS-CoV-2, since a synergistic action with other factors (such as meteorological, socio-economic and cultural factors) could not be excluded by the present study.

**Supplementary Information:**

The online version contains supplementary material available at 10.1007/s11356-021-12735-x.

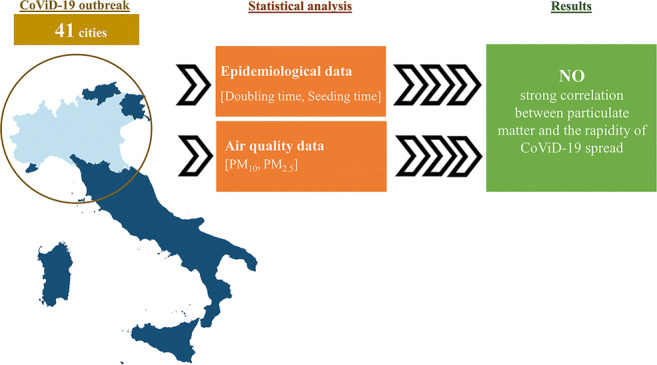

## Introduction

A strong correlation between air particulate pollution and the increase of autoimmune and respiratory diseases has been confirmed by several studies (Cruz-Sanchez et al. 2013; Horne et al. 2018; Tateo et al. 2019; Xu et al. 2016; Zhou et al. 2015). Moreover, recent studies highlighted a positive correlation between the mortality rate for CoViD-19 and long-term exposure to high concentrations of pollutants such as particulate matter (PM), SO_2_, CO, NO_2_ and O_3_ (Coccia 2020; Ogen 2020; Coker et al. 2020; Wu et al. 2020a, 2020b; Perone 2021; Yari and Moshammer 2020). For instance, Cole et al. (2020) observed a link between long-term PM_2.5_ exposure and CoViD-19 cases, hospital admissions and deaths in 355 municipalities in the Netherlands. Moreover, Isphording and Pestel (2020) statistically analysed the proliferation and aggressiveness of CoViD-19 in Germany highlighting a strong correlation between short-term exposure to air pollution and severe clinical reactions. However, the air pollution may have influenced not only the aggressiveness of CoViD-19.

The Italian Society of Environmental Medicine (SIMA) (SIMA 2020) supposed for the first time a possible correlation between the significant spread of coronavirus disease (CoViD-19), caused by Severe Acute Respiratory Syndrome Coronavirus 2 (SARS-CoV-2) (Collivignarelli et al. 2020b), in Northern Italy and the high levels of PM_10_ and PM_2.5_. Setti et al. (2020a, 2020b) and several other authors supposed that PM could acts as a support for novel SARS coronavirus (SARS-CoV-2), allowing the spread and the transport even for significant distances. According to this thesis, PM could represent a substrate that allows the virus to remain in the air in a contagious form for hours or days, promoting its diffusion (Sanità di Toppi et al. 2020; SIMA 2020). On the contrary, Belosi et al. (2021) showed that the probability of transmission in the outdoor environment of SARS-CoV-2 is not subjected to a substantial increase even in the presence of a high concentration of PM. This would seem to deny the presence of a possible strong correlation between the high rapidity of CoViD-19 spread in some areas of northern Italy and atmospheric PM. Therefore, due to contrasting results observed, this aspect is still the subject of analysis and discussion by the scientific community.

To date, no studies evaluated whether PM_10_ and PM_2.5_ influence the diffusion rapidity of the virus (intended as doubling time and seeding time (DT and ST), respectively), playing or not a key role in the massive spread of CoViD-19. This study aims to explore this relationship in Northern Italy, which has been the most affected area by CoViD-19 (INCP 2020), and is also the portion of the country presenting some areas with the highest amount of atmospheric PM often exceeding the legislative limit (Ionescu et al. 2013; Torretta et al. 2013; Masiol et al. 2015). Together with Poland and Bulgaria, Northern Italy has the worst air quality in Europe in terms of PM (EEA 2019, 2018, 2017). Data of PM_10_ and PM_2.5_ were analysed and, considering an incubation time of 10–15 d, were compared with the CoViD-19 rapidity of spread, evaluated using the ST and DT, in 41 cities of Northern Italy. In order to compare air quality data with epidemiological data, a statistical analysis was conducted and also the CoViD-19 ST/DT model proposed by Zhou et al. (2020) for the evaluation of the epidemic risk was used to investigate a possible correlation with PM.

## Methods

### Area of the study

**Fig. 1:**
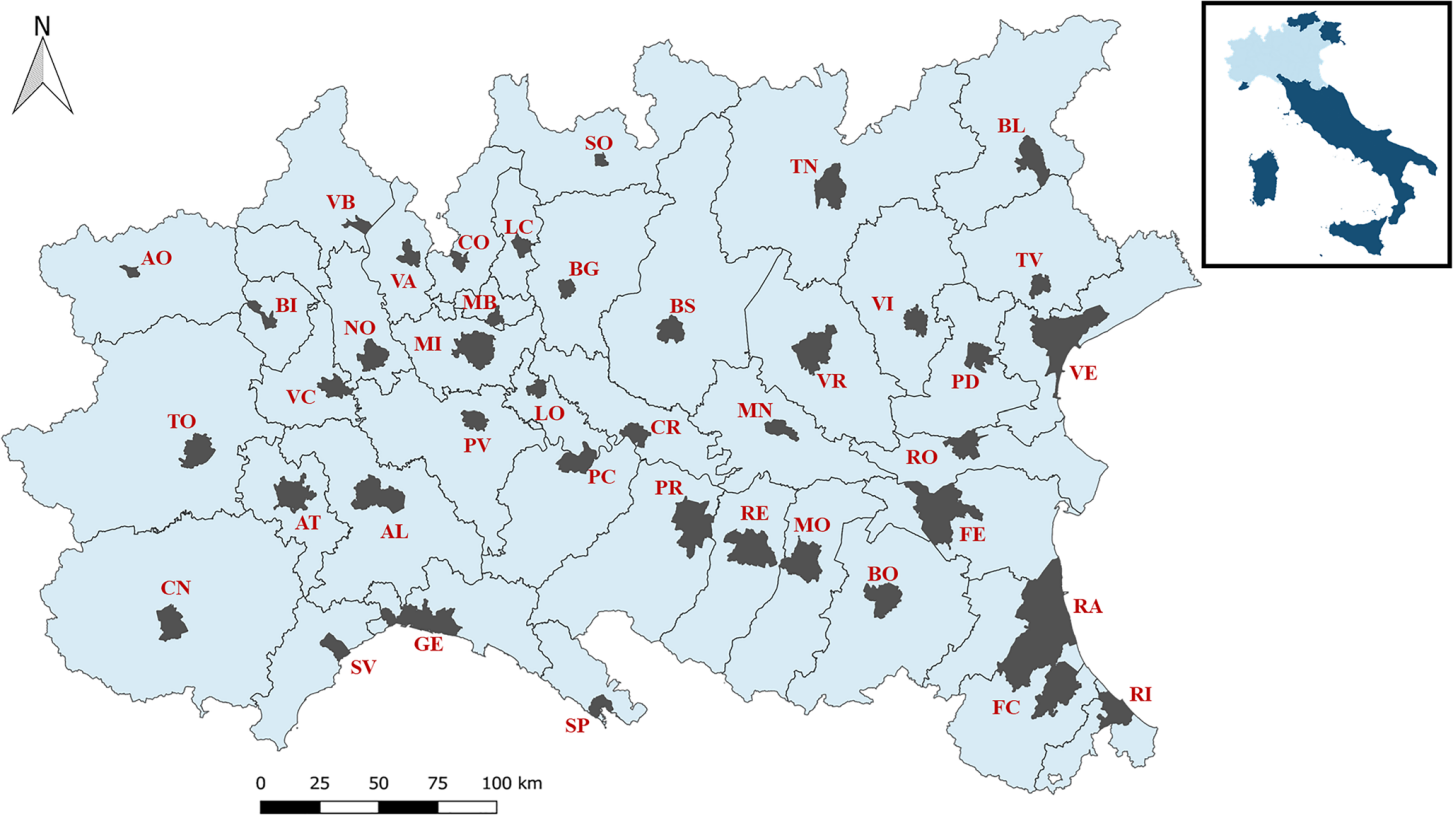
Map of the area analysed in the study and its location in Italy. The capital city for each province is highlighted in grey. The map has been realised with QGIS (2020). AL: Alessandria; AO: Aosta; AT: Asti; BG: Bergamo; BI: Biella; BL: Belluno; BO: Bologna; BS: Brescia; CN: Cuneo; CO: Como; CR: Cremona; FC: Forlì and Cesena; FE: Ferrara; GE: Genoa; LC: Lecco; LO: Lodi; MB: Monza; MI: Milan; MN: Mantova; MO: Modena; NO: Novara; PC: Piacenza; PD: Padua; PR: Parma; PV: Pavia; RA: Ravenna; RE: Reggio Emilia; RI: Rimini; RO: Rovigo; SO: Sondrio; SP: La Spezia; SV: Savona; TN: Trento; TO: Turin; TV: Treviso; VA: Varese; VB: Verbania; VC: Vercelli; VE: Venice; VI: Vicenza; VR: Verona

Considering that CoViD-19 has broken out in Northern Italy, this part of the country has been selected in order to detect a possible correlation between epidemic spread and PM in air. According to the most recent available data, the area of the study was larger than 100,000 km^2^ (ISTAT 2020a) and divided in 41 provinces, in seven different Regions (Piedmont, Valle d’Aosta, Lombardy, Liguria, Veneto, Trentino and Emilia-Romagna) totally accounting for around 25.8 million of inhabitants (ISTAT 2020b). The analysis has been applied on the capital of each province. In Fig. 1, the map of the selected provinces and the location of capital cities are reported.

### Epidemiological data collection and processing

The epidemiological evolution of the new SARS-CoV-2 cases was not available at city level. Therefore, the epidemiological data referred to each single province, provided by the Italian National Civil Protection (INCP 2020), were considered. In order to compare the spread rapidity of the CoViD-2019, seeding time (ST) and doubling time (DT) were used. As proposed by Zhou et al. (2020), two authors independently selected the date on which each epidemic curve seemed to rise and, in case of discrepancy, a third author defined which of the two dates to choose. The median of the cumulative cases reported up to the day before the rise of the curve (T1) was called seeding number (SN). The ST was considered equivalent to the time that elapses between the first case identified in each province and reported by official data (T0) and the achievement of a number of cases equal to SN. To quantify the DT, the new cumulative cases from T1 were fitted with exponential curve (Eqs. (1) and (2)):

1 $$ I={I}_0+{a}_1\ast {e}^{t/a2} $$

2 $$ \mathrm{DT}\ \left[d\right]={a}_2\ast \ln (2) $$

where *I* represents the number of new infections and *t* [*d*] is the progressive number of days (considering T1 as the first day).

### Particulate matter data collection and processing

Data of PM_10_ and PM_2.5_, for all cities, were collected from the local environmental protection agencies (APPA Trento 2020; ARPA Emilia-Romagna 2020; ARPA Liguria 2020; ARPA Lombardia 2020; ARPA Piemonte 2020; ARPA Valle d’Aosta 2020; ARPA Veneto 2020). All air quality control units, located in the capital cities, which measured PM in the selected periods, were used (Table S1) in order to obtain PM_10_ and PM_2.5_ concentrations. Forlì and Cesena are co-capitals of their province and were considered as a single city. Data of PM_2.5_ in Belluno, Ferrara and Reggio Emilia were not available. The daily averages (24 h) of the air pollutants for each city were calculated with the median, the standard deviation and the confidence interval.

### Comparison of the data

In order to compare air quality data (PM_10_ and PM_2.5_) with epidemiological data (ST and DT), a statistical analysis was conducted, and the correlation matrices of Pearson and Spearman were identified. Moreover, three different fittings (linear, quadratic and cubic) were used to investigate a correlation between the CoViD-19 spread rapidity in Northern Italy and PM. Finally, the CoViD-19 model, already proposed by Zhou et al. (2020) for the evaluation of the epidemic risk of a given area based on the DT and the ST, was used and the results were compared with the concentration of PM to investigate the possible influence on CoViD-19 spread rapidity.

### Determination of periods

**Fig. 2:**
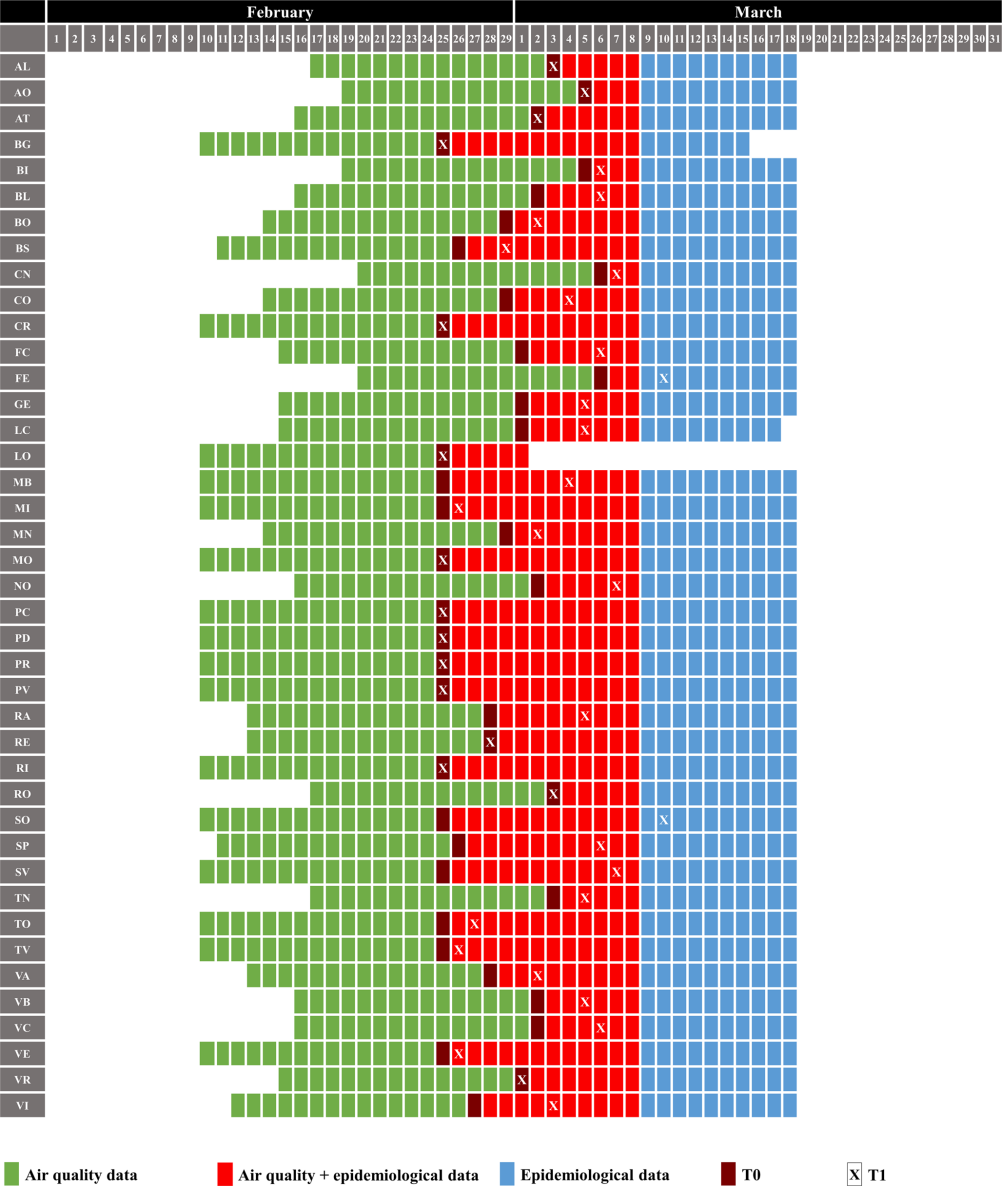
Selected periods for air quality monitoring and epidemiological data collection. T0 represents the first case identified in each province and reported by official data and T1 the day before the rise of the epidemiological curve. For the determination of T0 and T1, please refer to “Epidemiological analysis”. AL: Alessandria; AO: Aosta; AT: Asti; BG: Bergamo; BI: Biella; BL: Belluno; BO: Bologna; BS: Brescia; CN: Cuneo; CO: Como; CR: Cremona; FC: Forlì and Cesena; FE: Ferrara; GE: Genoa; LC: Lecco; LO: Lodi; MB: Monza; MI: Milan; MN: Mantova; MO: Modena; NO: Novara; PC: Piacenza; PD: Padua; PR: Parma; PV: Pavia; RA: Ravenna; RE: Reggio Emilia; RI: Rimini; RO: Rovigo; SO: Sondrio; SP: La Spezia; SV: Savona; TN: Trento; TO: Turin; TV: Treviso; VA: Varese; VB: Verbania; VC: Vercelli; VE: Venice; VI: Vicenza; VR: Verona

The choice of the periods in which to select the epidemiological and the air quality data has been made considering the average incubation time of the SARS-CoV-2 to evaluate the actual period during which contagion among people could have occurred. Several studies determined that the incubation time could be up to 10–15 d (Backer et al. 2020; Lai et al. 2020; Li et al. 2020). Therefore, the air quality data have been selected anticipating by 15 d the T0 and ending, as a maximum precautionary limit, in the 8th March 2020 (for the determination of T0, please refer to “Epidemiological analysis”). In the 8th March 2020, several restrictions were imposed in part of Northern Italy, and in 9th March, 2020, they were extended at the rest of the country (DPCM 2020a, b). Following the further increase in the number of infections, the restrictions were made even more severe starting from March 11th, 2020 (DPCM 2020c). In order to study only the exponential tract of the contagion curve, no epidemiological data after the 18th March 2020 were considered (Fig. 2). In some red areas (around the city of Lodi), the restrictions have been imposed earlier than in the rest of the region. The different lockdown timing could have direct repercussions on the epidemiological curve, and therefore in Lodi, the selected period ended in 1 March 2020 (IMH 2020a, 2020b). Also in Bergamo and Lecco, the influence due to lockdown was already visible before the 18th March 2020. In these cases, the selected periods were shortened.

## Results and discussion

### Epidemiological analysis

**Fig. 3:**
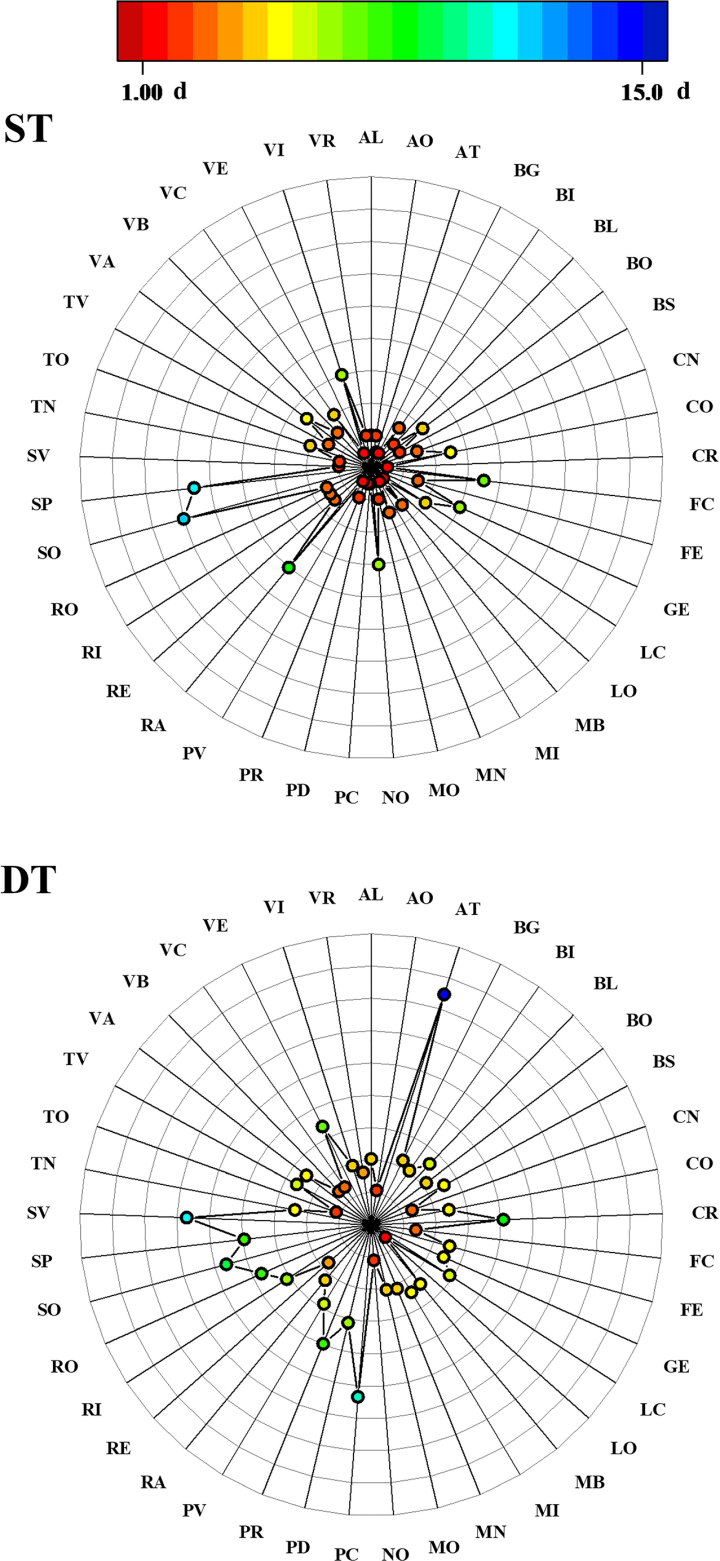
Value of seeding time (ST) and doubling time (DT) for each city. AL: Alessandria; AO: Aosta; AT: Asti; BG: Bergamo; BI: Biella; BL: Belluno; BO: Bologna; BS: Brescia; CN: Cuneo; CO: Como; CR: Cremona; FC: Forlì and Cesena; FE: Ferrara; GE: Genoa; LC: Lecco; LO: Lodi; MB: Monza; MI: Milan; MN: Mantova; MO: Modena; NO: Novara; PC: Piacenza; PD: Padua; PR: Parma; PV: Pavia; RA: Ravenna; RE: Reggio Emilia; RI: Rimini; RO: Rovigo; SO: Sondrio; SP: La Spezia; SV: Savona; TN: Trento; TO: Turin; TV: Treviso; VA: Varese; VB: Verbania; VC: Vercelli; VE: Venice; VI: Vicenza; VR: Verona

The official number of infections has been used to determine T0 and T1 for each province (Table S2), and fitting the cumulative number of total infections from T1 by an exponential curve (Fig. S1 and Tab. S3), the ST and DT have been calculated for each city considered in the study (Fig. 3). These values varied significantly.

Regarding the ST, the areas that have values equal to 1, and which a surge in infections has been highlighted right from the start, are those that showed the first clusters of CoViD-19 (mainly south—Lombardy and Padua in Veneto). Other provinces, on the other hand, showed an effective growth of the epidemic curve only several days after the first recorded cases (e.g. 12 days in Sondrio, 11 days in La Spezia, 8 days in Ravenna and 5 days in Como and Varese).

Asti and Savona showed the higher DT, 14.9 d and 11.4 d, respectively, representing a slower spread of the CoViD-19 infection among the population. On the contrary, other cities such as Lodi, Aosta, Novara and Turin were characterised by a DT fewer than 2.5 d. In these cases, the transmission of the virus among the population was faster. As expected, the minor DT (1.2 d) belongs to Lodi which was the first area of contagion and outbreak of the CoViD-19 in Italy. The DT obtained in this study is in accordance with other results reported in scientific literature. D’Arienzo and Coniglio (2020) identified DT equals to 3.1 d for Italy in the period February 25th–March 12th, 2020. In the period February 20th–March 24th, in the Italian regions of Lombardy and Emilia Romagna, Riccardo et al. (2020) evaluated DT equals to 2.7 d. Setti et al. (2020b) highlighted that in Milan, before March 13th, DT was 2.0 d.

### Particulate matter

**Fig. 4:**
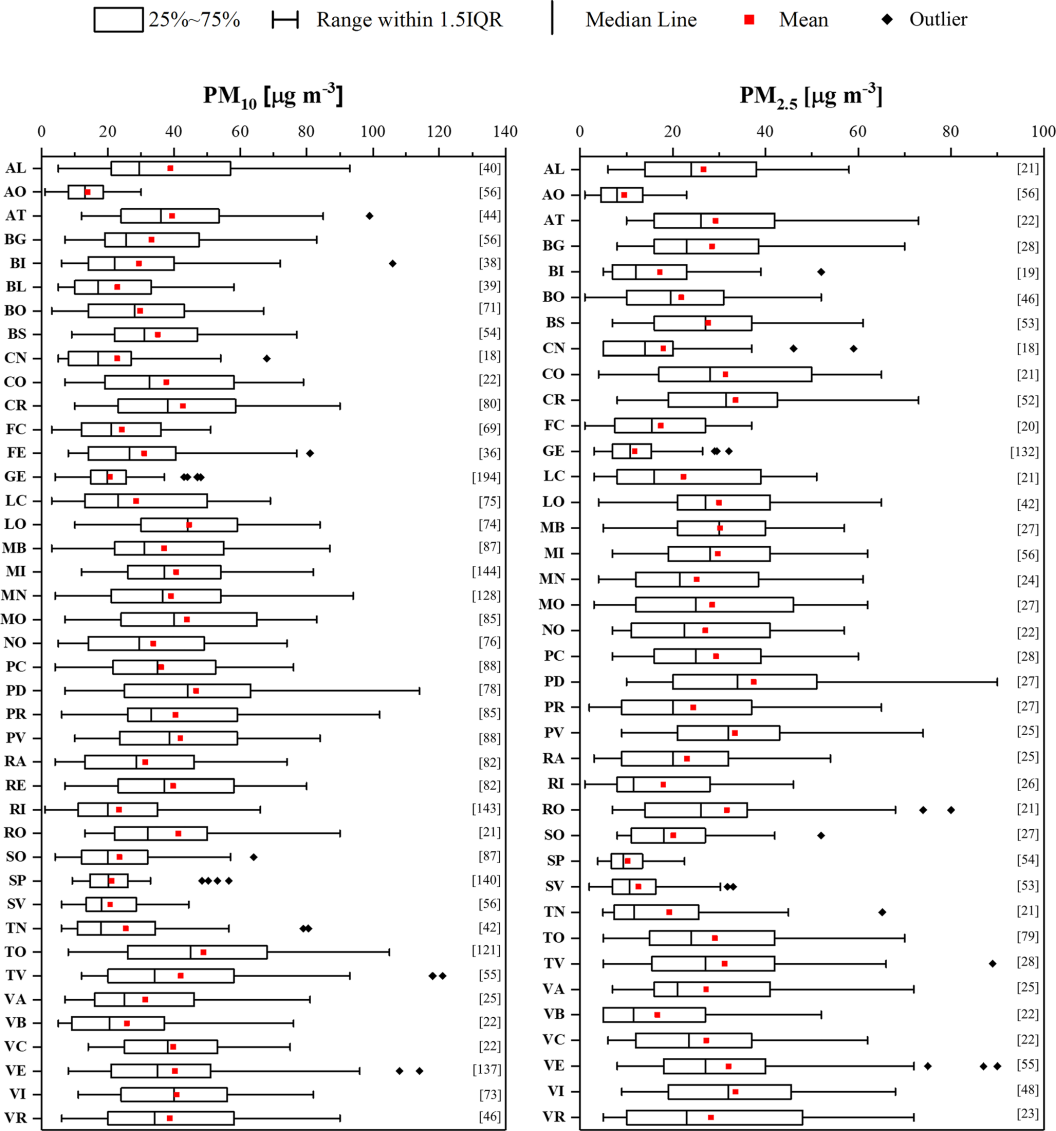
PM_10_ and PM_2.5_ concentrations during air quality monitoring period for each city. In brackets, numbers of data are reported. Boxplots represent the distance between the first and third quartiles while whiskers are set as the most extreme (lower and upper) data point not exceeding 1.5 times the quartile range from the median. AL: Alessandria; AO: Aosta; AT: Asti; BG: Bergamo; BI: Biella; BL: Belluno; BO: Bologna; BS: Brescia; CN: Cuneo; CO: Como; CR: Cremona; FC: Forlì and Cesena; FE: Ferrara; GE: Genoa; LC: Lecco; LO: Lodi; MB: Monza; MI: Milan; MN: Mantova; MO: Modena; NO: Novara; PC: Piacenza; PD: Padua; PR: Parma; PV: Pavia; RA: Ravenna; RE: Reggio Emilia; RI: Rimini; RO: Rovigo; SO: Sondrio; SP: La Spezia; SV: Savona; TN: Trento; TO: Turin; TV: Treviso; VA: Varese; VB: Verbania; VC: Vercelli; VE: Venice; VI: Vicenza; VR: Verona

In Fig. 4, the average and median values of PM_10_ and PM_2.5_ in each city for the selected periods are shown. Among the 41 cities, the situation was very heterogeneous. Cremona, Lodi, Milan, Modena, Padua, Parma, Pavia, Rovigo, Turin, Treviso, Venice and Vicenza presented a mean value of PM_10_ above 40 μg m^−3^ and the highest mean value of PM_10_ (48.8 μg m^−3^) was reached in Turin. A similar trend was observed for PM_2.5_, where a concentration higher than 30 μg m^−3^ was found in Cremona, Lodi, Monza, Padua, Pavia, Rovigo, Treviso, Venice and Vicenza. In this case, Padua showed the highest mean value of PM_2.5_ (37.4 μg m^−3^). On the contrary, the lowest mean values of PM_10_ and PM_2.5_ were detected in Aosta and were equal to 13.8 μg m^−3^ and 9.5 μg m^−3^, respectively. Other areas with the low mean values of PM_10_ and PM_2.5_ were the seaside cities of Genoa (20.6 μg m^−3^ and 11.8 μg m^−3^, respectively), Savona (20.6 μg m^−3^ and 12.5 μg m^−3^, respectively) and La Spezia (21.2 μg m^−3^ and 10.3 μg m^−3^, respectively), also in this case probably due to weather conditions, such as wind and precipitation, that could have positively influenced the air quality. In fact, the concentration of atmospheric PM is highly sensitive to weather conditions and factors such as wind and precipitation can strongly influence its concentration in the air (Baklanov et al. 2016; Collivignarelli et al. 2020a).

### Statistical analysis and discussion

To assess a possible dependence between the rapidity of spread of CoViD-19 among the population and the concentration of PM_10_ and PM_2.5_, air quality and epidemiological data were analysed and Pearson and Spearman correlations were calculated (Table 1). The results show that Spearman’s *R* is higher for DT-PM_10_ and DT-PM_2.5_: 0.0436 and 0.2361, respectively. Pearson’s and Spearman’s *R* are substantially equal for ST-PM_10_ and ST-PM_2.5_. Moreover, as confirmed by the literature (Andrée 2020), a very strong positive correlation between PM_10_ and PM_2.5_ exists (*R*= 0.9122 and 0.8667 with Pearson and Spearman, respectively). It is however evident that the correlation indices between PM and the DT of the number of infected people (inversely proportional to propagation speed of the epidemic) are substantially very low. The results highlighted also low negative values of Pearson and Spearman correlation indices between PM and the ST (also in this case, inversely proportional to the rapidity of CoViD-19 spread). The fact that ST and DT are unrelated to each other (both for Pearson and for Spearman) represent an aspect that highlights how these two parameters are strictly influenced by other aspects, such as sociability and living conditions, resulting from the presence of the coronavirus.

**Table 1:** Pearson and Spearman correlations for DT, ST, PM_10_ and PM_2.5_.

		DT	ST	PM_10_	PM_2.5_
DT	Pearson	1			
	Spearman	1			
ST	Pearson	−0.0398^b^	1		
	Spearman	−0.2307^a^	1		
PM_10_	Pearson	0.0065^b^	−0.3887^a^	1	
	Spearman	0.0436^b^	−0.3785^a^	1	
PM_2.5_	Pearson	0.0646^b^	−0.3969^a^	0.9122^b^	1
	Spearman	0.2361^a^	−0.3990^a^	0.8667^a^	1

^a^*p*-value >0.05.

^b^*p*-value < 0.05

**Fig. 5:**
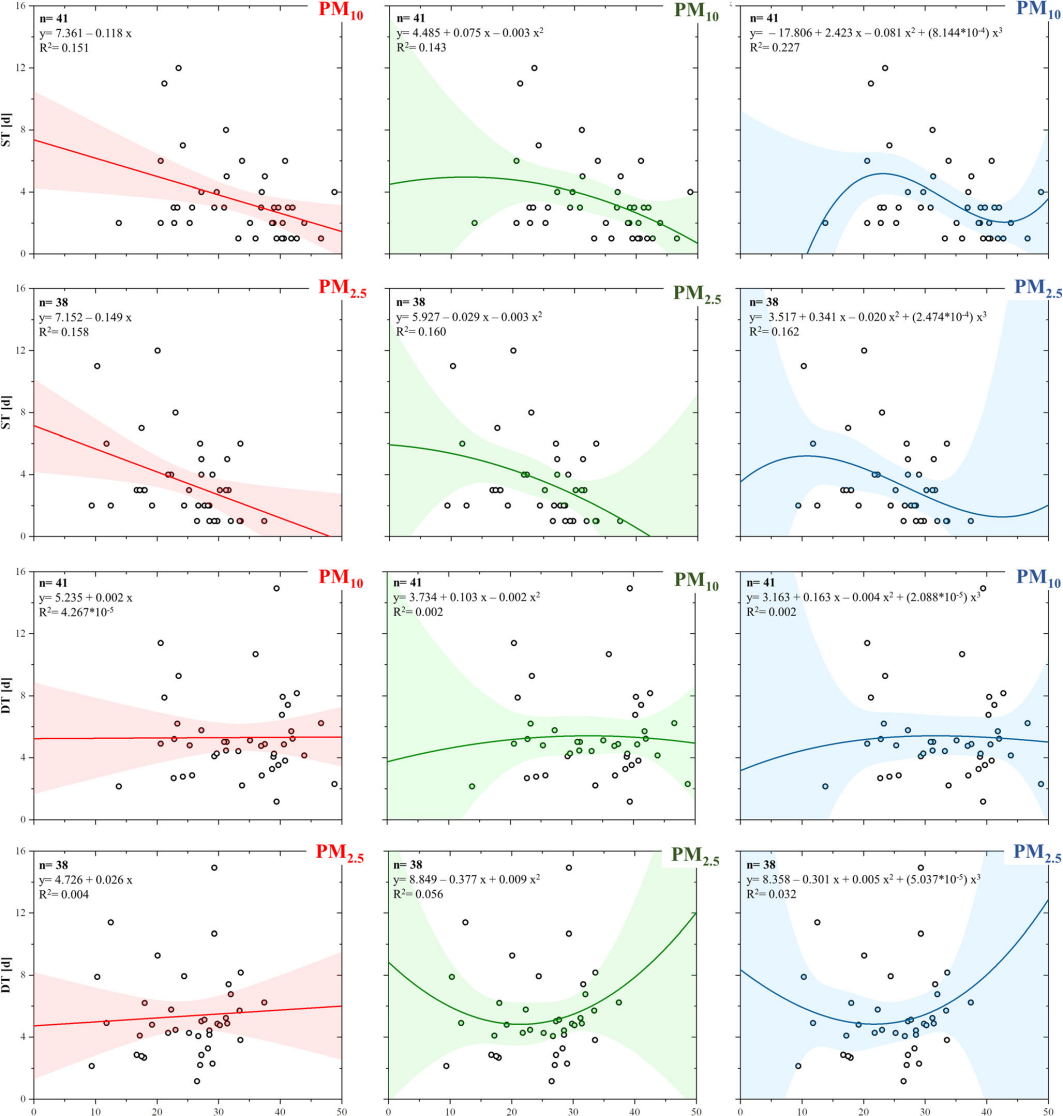
Fitting of ST and DT with PM_10_ and PM_2.5_ with linear (red), parabolic (green) and cubic (blue) functions. The coloured bands represent the 95% confidence interval. *n*: number of data

In order to better study the behaviour of ST and DT as a function of PM_10_ and PM_2.5_, these values have been fitted with linear, quadratic and cubic functions (Fig. 5). In the ST-PM_10_ case, the function that best approximates the points is that of 3rd degree (*R*^2^ = 0.227), while in the case of ST-PM_2.5_, all functions return substantially equal *R*^2^ (0.158–0.162). In the DT-PM_10_ and DT-PM_2.5_ analysis, the *R*^2^ remained always nearly to zero. In all cases, two aspects can be highlighted: (i) the 2nd- and 3rd-degree functions do not substantially improve the fitting of the points with respect to the linear function and (ii) the *R*^2^ values remain decidedly low in order to define the accurate fitting.

**Fig. 6:**
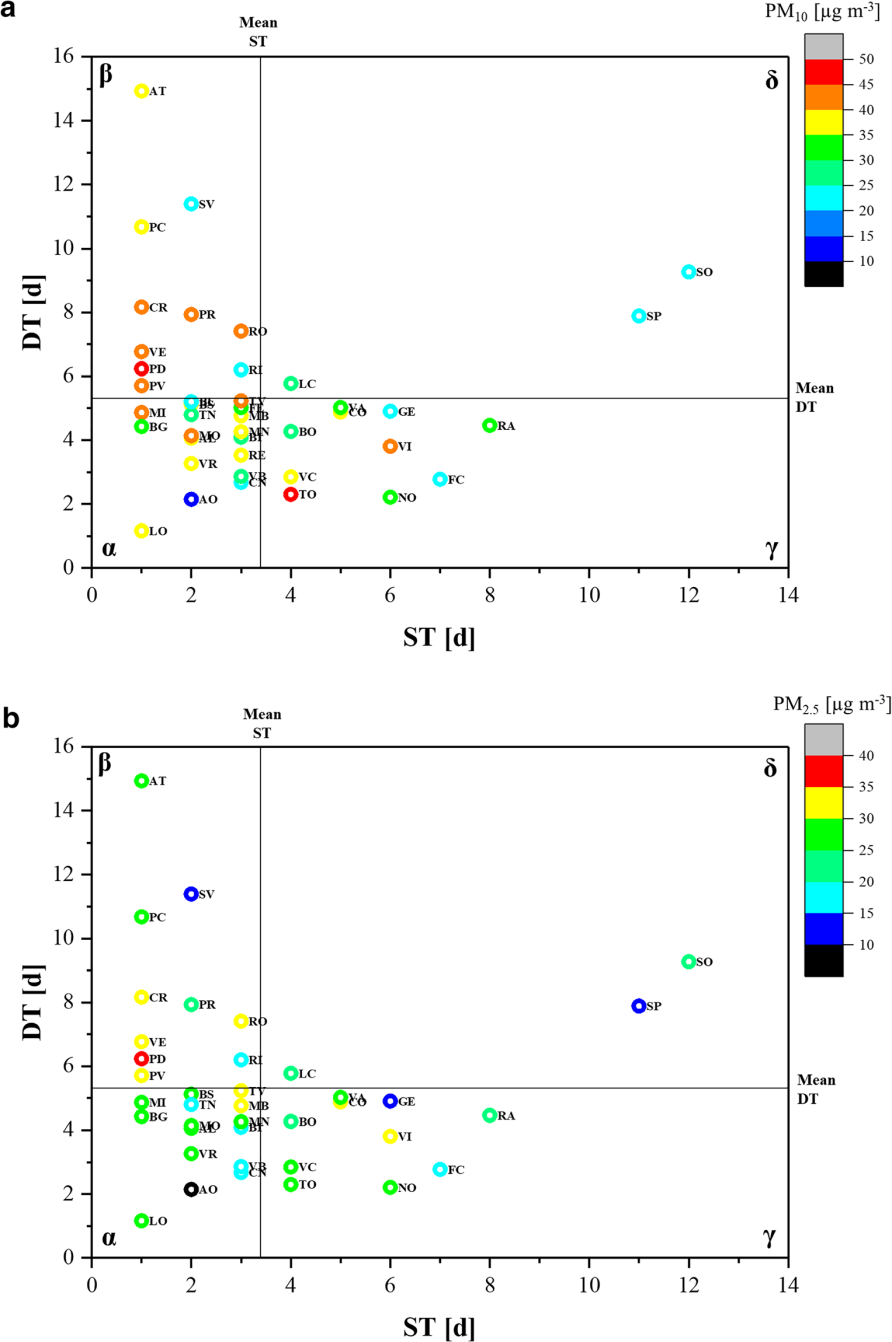
Map of the risk using the ST/DT model proposed by Zhou et al. (2020). α: high risk; β: moderately high risk; γ: moderately low risk; δ: low risk

Zhou et al. (2020) proposed a model for the assessment of epidemic risk in different countries of the world determining four risk categories (high risk, moderately high risk, moderately low risk and low risk) based on the values of ST and DT. This model has been applied on the data processed in the current study, and the results were compared with the mean concentration of PM to investigate a possible correlation (Fig. 6).

**Fig. 7:**
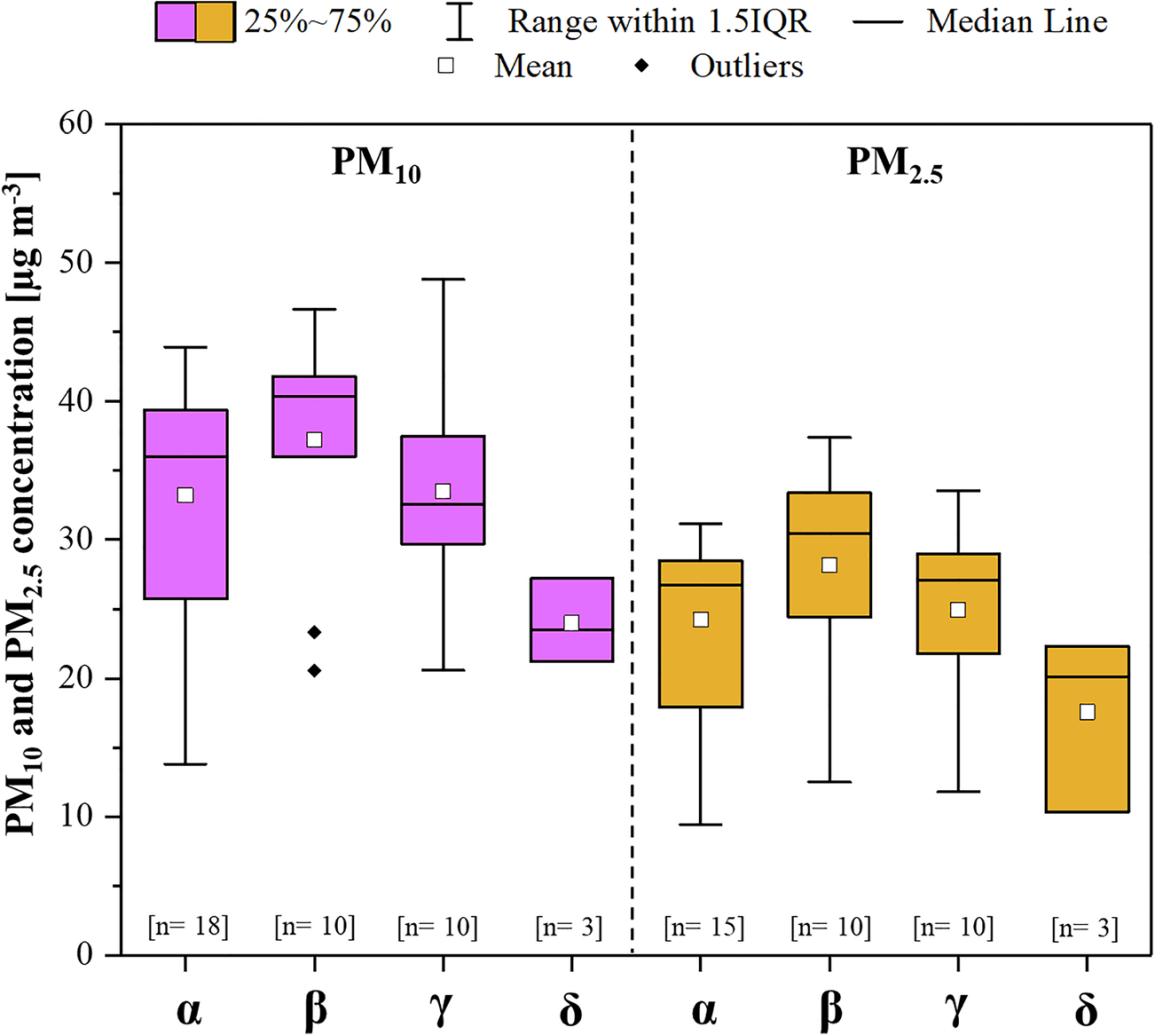
PM_10_ and PM_2.5_ concentrations by risk bands. Boxplots represent the distance between the first and third quartiles while whiskers are set as the most extreme (lower and upper) data point not exceeding 1.5 times the quartile range from the median. *n*: number of data; α: high risk; β: moderately high risk; γ: moderately low risk; δ: low risk

According to the model of Zhou et al. (2020), the results show that almost all the cities in Northern Italy analysed were in the high, moderately high, and moderately low risk bands in the initial phase of CoViD-19 spread. In the low epidemic risk band, the measured PM_10_ concentration was slightly lower than in the other bands. However, this last result should be considered partial given the limited sample size (*n* = 3). Comparing the PM concentrations in the cities located in the other bands, no substantial differences were highlighted. For instance, the average concentration of PM_10_ and PM_2.5_ in the areas identified at moderately low risk was slightly higher than that measured in the areas considered at high risk (Fig. 7). Therefore, also using the risk model proposed by Zhou et al. (2020), no strong influence of PM on CoViD-19 spread rapidity can be highlighted.

The strength of this study lies in immediacy and practicality. Comparing the epidemiological and air quality data of 41 cities in Northern Italy in the most affected regions from CoViD-19 and taking also into account the possible incubation period of the disease, the results allowed excluding that high concentration of PM was the primary cause of the high rapidity of CoViD-19 spread. This agrees with what Belosi et al. (2021) found. By estimating the concentration of SARS-CoV-2 in outdoor air in Lombardy using a box model, they excluded that the presence of high concentrations of PM could act as a vehicle for the infection (Belosi et al. 2021).

However, the opinion of the scientific community on the influence of PM on the infected population rate is conflicting. Other studies obtained positive correlations among the number of infected, mortality, and the concentration of PM in the air (Ma et al. 2020; Zhu et al. 2020). For instance, Setti et al. (2020b) would seem to have obtained an opposite result compared to the present study, highlighting a clear correlation between PM and SARS-CoV-2 infections. However, the results of the present study are only partially comparable with literature (e.g., Setti et al. (2020b)), where in most cases the correlation between PM and the rate of infected people (cases/population) was assessed, instead of the rapidity of contagion with the use of DT. This difference in result could be attributed to different methodological approaches and also to different scopes. In fact, the present paper aims to evaluate the possible primary role of PM in the rapidity of virus transmission rather than investigating the influence of average daily PM_10_ exceedances on the rate of infections. Also, Delnevo et al. (2020) observed that a possible statistical correlation between air pollution and CoViD-19 infections, in Emilia-Romagna (Italy), could exist. However, even in this case, the results are only partially comparable with the present study as the new daily infections were used to evaluate the epidemiological situation, instead of the rapidity of contagion intended as DT.

Despite the results of the present study exclude that PM alone was the primary the high CoViD-19 spread rapidity in some areas of Northern Italy, it is becoming increasingly clear, also by following the epidemic trends worldwide, that several other aspects should also be considered. Focusing exclusively on air pollution can lead to spurious associations, because socio-economic and cultural factors often play a concurrent role. Andree (2020) presents a detailed analysis of the Dutch situation, by studying areas, hotspots, pollution loads, social links, habits, age, gender, household composition and lifestyle. In addition, the variability in healthcare systems and identification practises of infected people highly affect the data about pandemic behaviour. Liu et al. (2020) observed that also meteorological factors play a role in the CoViD-19 transmission and SARS-CoV-2 transmission was likely favoured by low temperature, mild diurnal temperature range and low humidity. Therefore, a synergistic action of PM with other factors, e.g., other air pollutants, meteorological conditions and socio-economic aspects, could not be excluded.

## Discussion about the possible limitations of the present study

These results should be considered in the light of some possible limitations. In this study, the Authors did not consider the synergistic action of PM with other factors. As for other viral pathogens, high particulate concentration plays a crucial role in weakening the immune system (Glencross et al. 2020). In case of chronic exposure, the atmospheric particulate has proved to indirectly promote the diffusion of SARS-CoV-2, e.g., by enhancing its adhesion to angiotensin-converting enzyme 2 (ACE2) (Comunian et al. 2020; Tung et al. 2021). Together with particulate matter, the long-term exposure to other atmospheric pollutants increases the susceptibility not only to respiratory viral (and bacterial) pathogens but also, exerting a chronic inflammatory stimulus, to cardiovascular and neoplastic diseases (Andersen et al. 2017; Kim et al. 2017; Yang et al. 2019; Conticini et al. 2020; Fattorini and Regoli 2020; Iriti et al. 2020). These represent co-morbidity factors in case of CoViD-19 (Sanyaolu et al. 2020). Indeed, several other authors considered also the parallel contribution of other socio-economic aspects (e.g., density and age of population, mobility of population and healthcare expenditures) (e.g., Andree 2020). However, our goal was assessing the possible primary role of PM in SARS-CoV-2 transmission in Northern Italy, hence in epidemic spread, rather than investigating the severity of cases. In fact, in this study also, the results of swabs of asymptomatic subjects have been counted.

Failure to consider social distancing measures adopted by Italian population independently from Government actions could represent another limitation of this work because this could make difficult to compare the differences in DTs. However, to overcome this aspect focusing on the massive increase occurred before the blockade was imposed, the authors neglected the data (i.e., the number of positive swabs) of the period in which the effects of the lockdown started to be visible.

Moreover, the possibility that a person residing in one province may be infected in another following a travel for work, study or leisure purposes was also not considered and could represent a limitation of the study. Mobility between provinces and regions is an aspect that could be studied in the future by adopting an estimate of the mobility rate despite this being opposed to the immediacy and practicality of the current model.

Finally, the geographical area that the authors chose can appear slightly exiguous knowing that the use of macroscopic statistical models to small territories involves a great variability. In the initial phase of CoViD-19 spread, Italy was almost divided in two parts, being Centre and South all but free from infection nuclei. Therefore, including a broader area, beside North, would have led to inaccuracy. Northern Italy represented a grave and unique reality in Europe, because, there, first, the pandemics burst and grew exponentially. After this heavy onset, the lockdown national restrictions prescribed by the Government prevented the virus from spreading massively also in Central and Southern Italy. This scenery maintained “double” until the end of Italian first wave. Our criterion of data selection proved to be consistent again considering that in Centre and South the growth of the pandemic was detected after lockdown was imposed and therefore affected by the government measures. Northern Italy accounts for 34% of the Italian land and presents a huge heterogeneity in terms of PM concentration. The precise objective of the present investigation was to focus on the regions where people were heavily hit by the pandemics.

## Conclusion

In this work, considering an incubation time of 10–15 d, data on the concentration of PM_10_ and PM_2.5_ were analysed and compared with the rapidity of spread of CoViD-19 (intended as ST and DT) in 41 cities of Northern Italy. This work excludes that PM alone was the primary the high CoViD-19 spread rapidity. Pearson’s and Spearman’s indices did not highlight any correlation between PM and epidemiological data. Moreover, three different fittings (linear, quadratic and cubic) were used and in all cases of comparison two aspects can be highlighted: (i) the 2nd- and 3rd-degree functions do not substantially improve the fitting of the points with respect to the linear function and (ii) the *R*^2^ values remain decidedly low in order to define the accurate fitting. A model ST/DT for the evaluation of the epidemic risk, already proposed in literature, was applied but no strong influence of PM has been found. However, the authors do not exclude that a synergistic action with other factors (such as meteorological, socio-economic and cultural factors) could exist and therefore other studies to further comprehend, for instance, the influence of other atmospheric pollution parameters (e.g., NO_*x*_), meteorological conditions (e.g., temperature, solar irradiance and humidity), lifestyle and social links on the rapidity of spread of the SARS-CoV-2 are strongly suggested.

## Supplementary Information

ESM 1 (DOCX 456 kb)

ESM 2 (DOCX 19 kb)

ESM 3 (DOCX 38 kb)

ESM 4 (DOCX 29 kb)

## Data Availability

All data generated or analysed during this study are included in this published article
