# Supplementary material for: Can particulate matter be identified as the primary cause of the rapid spread of CoViD-19 in some areas of Northern Italy?
Source: Environ Sci Pollut Res Int. 2021 Feb 26;28(25):33120–32. doi: 10.1007/s11356-021-12735-x (PMC7909738; doi:10.1007/s11356-021-12735-x)
Supplement: Supplementary file 1 — (DOCX 456 kb) [file 11356_2021_12735_MOESM1_ESM.docx]

**Can particulate matter be identified as the primary cause of the rapid spread of CoViD-19 in some areas of Northern Italy?**

Maria Cristina Collivignarelli ^1,2^, Alessandro Abbà ^3^, Francesca Maria Caccamo ^1^, Giorgio Bertanza ^3^, Roberta Pedrazzani ^4^, Marco Baldi ^5^, Paola Ricciardi ^1^, Marco Carnevale Miino ^1,*^

^1^: Department of Civil Engineering and Architecture, University of Pavia, via Ferrata 3, 27100 Pavia, Italy

^2^: Interdepartmental Centre for Water Research, University of Pavia, via Ferrata 3, 27100 Pavia, Italy

^3^: Department of Civil, Environmental, Architectural Engineering and Mathematics, University of Brescia, via Branze 43, 25123 Brescia, Italy

^4^: Department of Mechanical and Industrial Engineering, University of Brescia, via Branze 38, I-25123, Brescia, Italy

^5^: Department of Chemistry, University of Pavia, viale Taramelli 10, 27100 Pavia, Italy

**^*^**: Corresponding author -> Email address: marco.carnevalemiino01@universitadipavia.it (Marco Carnevale Miino)

**Figure S1**


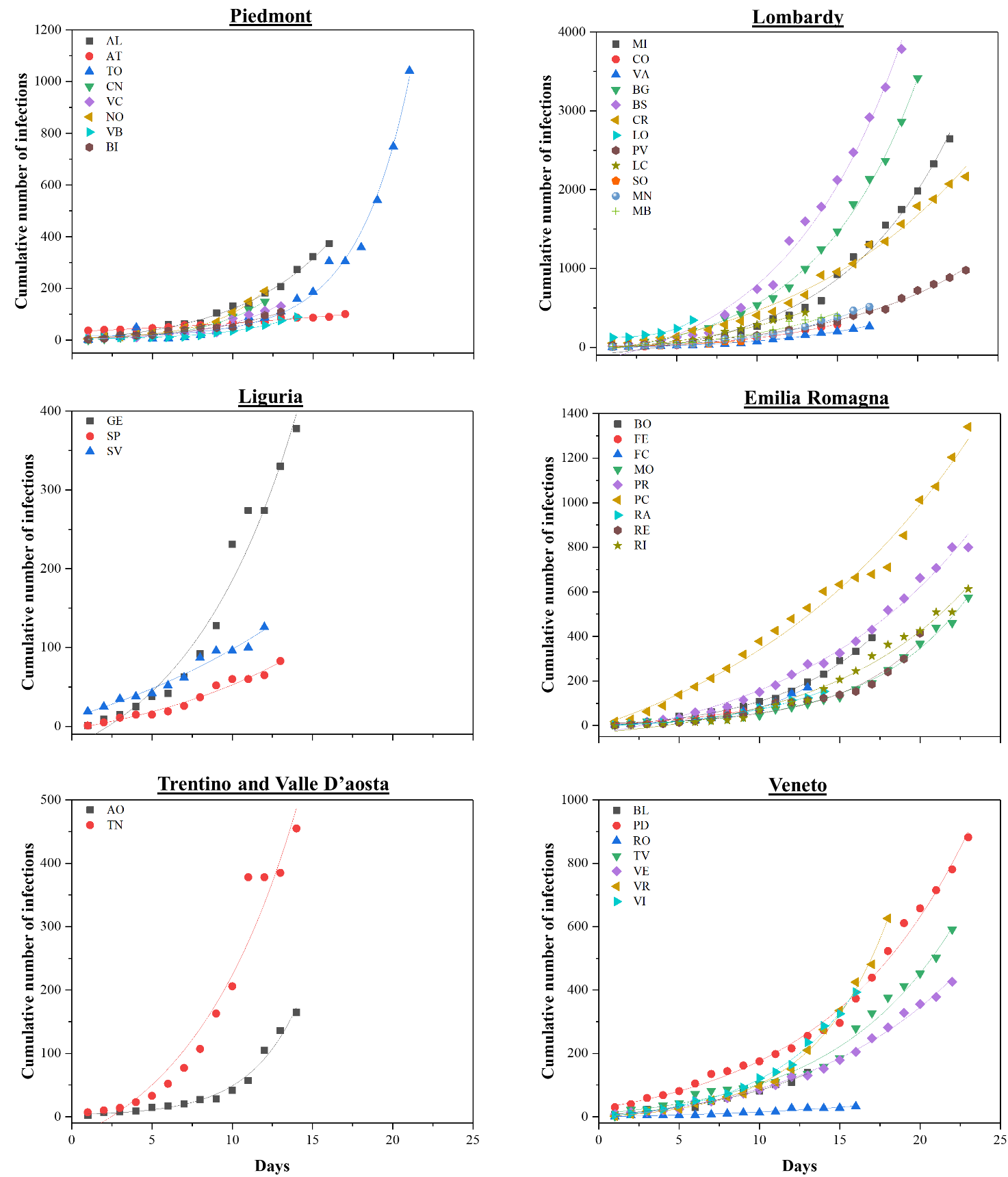


***Fig.S1*** *Epidemiological data from T1 and exponential fitted curves. AL: Alessandria; AO: Aosta; AT: Asti; BG: Bergamo; BI: Biella; BL: Belluno; BO: Bologna; BS: Brescia; CN: Cuneo; CO: Como; CR: Cremona; FC: Forlì and Cesena; FE: Ferrara; GE: Genoa; LC: Lecco; LO: Lodi; MB: Monza; MI: Milan; MN: Mantova; MO: Modena; NO: Novara; PC: Piacenza; PD: Padua; PR: Parma; PV: Pavia; RA: Ravenna; RE: Reggio Emilia; RI: Rimini; RO: Rovigo; SO: Sondrio; SP: La Spezia; SV: Savona; TN: Trento; TO: Turin; TV: Treviso; VA: Varese; VB: Verbania; VC: Vercelli; VE: Venice; VI: Vicenza; VR: Verona*
