# Supplementary material for: Can particulate matter be identified as the primary cause of the rapid spread of CoViD-19 in some areas of Northern Italy?
Source: Environ Sci Pollut Res Int. 2021 Feb 26;28(25):33120–32. doi: 10.1007/s11356-021-12735-x (PMC7909738; doi:10.1007/s11356-021-12735-x)
Supplement: Supplementary file 2 — (DOCX 19 kb) [file 11356_2021_12735_MOESM2_ESM.docx]

**Can particulate matter be identified as the primary cause of the rapid spread of CoViD-19 in some areas of Northern Italy?**

Maria Cristina Collivignarelli ^1,2^, Alessandro Abbà ^3^, Francesca Maria Caccamo ^1^, Giorgio Bertanza ^3^, Roberta Pedrazzani ^4^, Marco Baldi ^5^, Paola Ricciardi ^1^, Marco Carnevale Miino ^1,*^

^1^: Department of Civil Engineering and Architecture, University of Pavia, via Ferrata 3, 27100 Pavia, Italy

^2^: Interdepartmental Centre for Water Research, University of Pavia, via Ferrata 3, 27100 Pavia, Italy

^3^: Department of Civil, Environmental, Architectural Engineering and Mathematics, University of Brescia, via Branze 43, 25123 Brescia, Italy

^4^: Department of Mechanical and Industrial Engineering, University of Brescia, via Branze 38, I-25123, Brescia, Italy

^5^: Department of Chemistry, University of Pavia, viale Taramelli 10, 27100 Pavia, Italy

**^*^**: Corresponding author -> Email address: marco.carnevalemiino01@universitadipavia.it (Marco Carnevale Miino)

**Table S1** Number of air quality control units for PM_10_ and PM_2.5_ analysed in the study. a: Forlì and Cesena, co-capitals of their province, have been considered as a single city; b: The data refer to the air quality control unit located in Domodossola

| **City** | **Abbreviation** | **number of air quality control units analysed** | |
| --- | --- | --- | --- |
|  |  | **PM_10_** | **PM_2.5_** |
| Alessandria | AL | 2 | 1 |
| Aosta | AO | 3 | 3 |
| Asti | AT | 2 | 1 |
| Bergamo | BG | 2 | 1 |
| Biella | BI | 2 | 2 |
| Belluno | BL | 2 | 0 |
| Bologna | BO | 3 | 2 |
| Brescia | BS | 2 | 2 |
| Cuneo | CN | 1 | 1 |
| Como | CO | 1 | 1 |
| Cremona | CR | 3 | 2 |
| Forlì and Cesena ^a^ | FC | 3 | 1 |
| Ferrara | FE | 2 | 0 |
| Genova | GE | 9 | 6 |
| Lecco | LC | 2 | 1 |
| Lodi | LO | 2 | 2 |
| Monza | MB | 2 | 1 |
| Milano | MI | 4 | 2 |
| Mantova | MN | 4 | 1 |
| Modena | MO | 2 | 1 |
| Novara | NO | 2 | 1 |
| Piacenza | PC | 2 | 1 |
| Padova | PD | 3 | 1 |
| Parma | PR | 2 | 1 |
| Pavia | PV | 2 | 1 |
| Ravenna | RA | 2 | 1 |
| Reggio Emilia | RE | 2 | 0 |
| Rimini | RI | 4 | 1 |
| Rovigo | RO | 1 | 1 |
| Sondrio | SO | 2 | 1 |
| La Spezia | SP | 4 | 2 |
| Savona | SV | 2 | 2 |
| Trento | TN | 2 | 1 |
| Torino | TO | 5 | 3 |
| Treviso | TV | 2 | 1 |
| Varese | VA | 1 | 1 |
| Verbania | VB | 1 ^b^ | 1 ^b^ |
| Vercelli | VC | 2 | 1 |
| Venezia | VE | 5 | 2 |
| Vicenza | VI | 3 | 2 |
| Verona | VR | 2 | 1 |
