# Supplementary material for: Can particulate matter be identified as the primary cause of the rapid spread of CoViD-19 in some areas of Northern Italy?
Source: Environ Sci Pollut Res Int. 2021 Feb 26;28(25):33120–32. doi: 10.1007/s11356-021-12735-x (PMC7909738; doi:10.1007/s11356-021-12735-x)
Supplement: Supplementary file 3 — (DOCX 38 kb) [file 11356_2021_12735_MOESM3_ESM.docx]

**Can particulate matter be identified as the primary cause of the rapid spread of CoViD-19 in some areas of Northern Italy?**

Maria Cristina Collivignarelli ^1,2^, Alessandro Abbà ^3^, Francesca Maria Caccamo ^1^, Giorgio Bertanza ^3^, Roberta Pedrazzani ^4^, Marco Baldi ^5^, Paola Ricciardi ^1^, Marco Carnevale Miino ^1,*^

^1^: Department of Civil Engineering and Architecture, University of Pavia, via Ferrata 3, 27100 Pavia, Italy

^2^: Interdepartmental Centre for Water Research, University of Pavia, via Ferrata 3, 27100 Pavia, Italy

^3^: Department of Civil, Environmental, Architectural Engineering and Mathematics, University of Brescia, via Branze 43, 25123 Brescia, Italy

^4^: Department of Mechanical and Industrial Engineering, University of Brescia, via Branze 38, I-25123, Brescia, Italy

^5^: Department of Chemistry, University of Pavia, viale Taramelli 10, 27100 Pavia, Italy

**^*^**: Corresponding author -> Email address: marco.carnevalemiino01@universitadipavia.it (Marco Carnevale Miino)

***Table S2*** *Epidemiological data in the selected periods, from T0. The chosen T1 are highlighted in green. The data in orange and blue has been considered equal to 11 and 13, respectively. AL: Alessandria; AO: Aosta; AT: Asti; BG: Bergamo; BI: Biella; BL: Belluno; BO: Bologna; BS: Brescia; CN: Cuneo; CO: Como; CR: Cremona; FC: Forlì and Cesena; FE: Ferrara; GE: Genoa; LC: Lecco; LO: Lodi; MB: Monza; MI: Milan; MN: Mantova; MO: Modena; NO: Novara; PC: Piacenza; PD: Padua; PR: Parma; PV: Pavia; RA: Ravenna; RE: Reggio Emilia; RI: Rimini; RO: Rovigo; SO: Sondrio; SP: La Spezia; SV: Savona; TN: Trento; TO: Turin; TV: Treviso; VA: Varese; VB: Verbania; VC: Vercelli; VE: Venice; VI: Vicenza; VR: Verona*

|  | **AL** | **AT** | **TO** | **CN** | **VC** | **NO** | **VB** | **BI** | **AO** | **MI** | **CO** | **VA** | **BG** | **BS** | **CR** | **LO** | **PV** | **LC** | **SO** | **MN** | **MB** | **GE** | **SP** | **SV** | **BO** | **FE** | **FC** | **MO** | **PR** | **PC** | **RA** | **RE** | **RI** | **TN** | **BL** | **PD** | **RO** | **TV** | **VE** | **VR** | **VI** |  |
| --- | --- | --- | --- | --- | --- | --- | --- | --- | --- | --- | --- | --- | --- | --- | --- | --- | --- | --- | --- | --- | --- | --- | --- | --- | --- | --- | --- | --- | --- | --- | --- | --- | --- | --- | --- | --- | --- | --- | --- | --- | --- | --- |
| **1 = T0** | 1 | 37 | 3 | 1 | 1 | 3 | 4 | 2 | 2 | 8 | 1 | 3 | 18 | 2 | 53 | 125 | 27 | 2 | 1 | 1 | 3 | 1 | 1 | 1 | 2 | 1 | 1 | 3 | 4 | 18 | 1 | 1 | 1 | 4 | 3 | 30 | 1 | 1 | 7 | 2 | 3 |  |
| **2** | 16 | 40 | 3 | 1 | 1 | 3 | 4 | 3 | 7 | 8 | 2 | 4 | 20 | 10 | 57 | 128 | 27 | 3 | 3 | 4 | 4 | 1 | 1 | 10 | 2 | 2 | 1 | 8 | 8 | 28 | 1 | 4 | 3 | 5 | 5 | 40 | 4 | 2 | 8 | 8 | 3 |  |
| **3** | 22 | 41 | 2 | 5 | 3 | 3 | 5 | 6 | 8 | 15 | 2 | 4 | 72 | 13 | 91 | 159 | 36 | 4 | 3 | 5 | 5 | 1 | 1 | 18 | 3 | 6 | 1 | 18 | 10 | 63 | 2 | 7 | 6 | 7 | 7 | 59 | 5 | 22 | 14 | 17 | 3 |  |
| **4** | 32 | 43 | 11 | 11 | 8 | 3 | 5 | 19 | 9 | 29 | 4 | 4 | 103 | 14 | 123 | 182 | 49 | 5 | 3 | 15 | 6 | 1 | 1 | 18 | 6 | 7 | 2 | 18 | 27 | 89 | 2 | 8 | 9 | 10 | 7 | 68 | 5 | 23 | 15 | 21 | 3 |  |
| **5** | 40 | 47 | 11 | 14 | 7 | 4 | 9 | 18 | 15 | 30 | 5 | 7 | 110 | 49 | 136 | 237 | 55 | 8 | 3 | 22 | 6 | 1 | 1 | 41 | 11 | 8 | 4 | 22 | 35 | 138 | 2 | 14 | 15 | 14 | 7 | 81 | 5 | 35 | 19 | 25 | 3 |  |
| **6** | 60 | 50 | 49 | 17 | 10 | 5 | 10 | 20 | 17 | 46 | 11 | 11 | 209 | 60 | 214 | 344 | 78 | 11 | 3 | 26 | 6 | 9 | 1 | 19 | 19 | 12 | 3 | 24 | 59 | 174 | 2 | 20 | 16 | 23 | 11 | 105 | 5 | 43 | 24 | 42 | 5 |  |
| **7** | 63 | 58 | 6 | 24 | 15 | 13 | 13 | 36 | 20 | 58 | 11 | 17 | 243 | 86 | 223 |  | 83 | 35 | 3 | 32 | 8 | 15 | 1 | 16 | 41 | 17 | 7 | 29 | 61 | 212 | 3 | 31 | 19 | 33 | 23 | 135 | 7 | 72 | 48 | 52 | 10 |  |
| **8** | 65 | 58 | 7 | 40 | 11 | 14 | 11 | 39 | 27 | 93 | 23 | 23 | 372 | 127 | 287 |  | 122 | 53 | 3 | 46 | 9 | 25 | 1 | 20 | 49 | 24 | 15 | 33 | 84 | 256 | 8 | 44 | 24 | 52 | 29 | 144 | 10 | 82 | 48 | 63 | 19 |  |
| **9** | 105 | 58 | 11 | 47 | 18 | 22 | 11 | 48 | 28 | 145 | 27 | 27 | 423 | 155 | 333 |  | 126 | 66 | 4 | 56 | 11 | 38 | 1 | 19 | 62 | 29 | 16 | 41 | 115 | 319 | 10 | 48 | 33 | 77 | 29 | 162 | 14 | 86 | 59 | 73 | 24 |  |
| **10** | 132 | 68 | 19 | 61 | 24 | 27 | 13 | 48 | 42 | 197 | 40 | 32 | 537 | 182 | 406 |  | 151 | 89 | 4 | 102 | 19 | 42 | 1 | 20 | 80 | 34 | 20 | 45 | 150 | 378 | 13 | 70 | 68 | 107 | 30 | 175 | 13 | 89 | 73 | 96 | 37 |  |
| **11** | 136 | 69 | 34 | 91 | 25 | 32 | 18 | 50 | 57 | 267 | 46 | 44 | 623 | 413 | 452 |  | 180 | 113 | 4 | 119 | 20 | 63 | 5 | 15 | 86 | 44 | 24 | 73 | 181 | 426 | 19 | 103 | 93 | 163 | 48 | 198 | 16 | 103 | 85 | 110 | 50 |  |
| **12** | 182 | 70 | 55 | 119 | 29 | 48 | 29 | 67 | 105 | 361 | 77 | 50 | 761 | 501 | 562 |  | 221 | 199 | 6 | 137 | 61 | 92 | 11 | 19 | 108 | 58 | 33 | 82 | 229 | 479 | 24 | 104 | 104 | 206 | 59 | 216 | 27 | 110 | 100 | 150 | 53 |  |
| **13** | 207 | 87 | 89 | 149 | 31 | 48 | 36 | 96 | 136 | 406 | 98 | 75 | 997 | 739 | 665 |  | 243 | 237 | 6 | 169 | 59 | 128 | 15 | 25 | 122 | 64 | 49 | 97 | 276 | 528 | 31 | 114 | 113 | 378 | 78 | 255 | 27 | 126 | 126 | 210 | 73 |  |
| **14** | 273 | 87 | 87 |  | 84 | 71 | 50 | 109 | 165 | 506 | 118 | 98 | 1245 | 790 | 916 |  | 296 | 287 | 7 | 187 | 64 | 231 | 15 | 35 | 155 |  | 62 | 116 | 279 | 602 | 41 | 123 | 164 | 378 | 82 | 273 | 27 | 136 | 130 | 275 | 92 |  |
| **15** | 323 | 87 | 111 |  | 99 | 109 | 58 |  |  | 592 | 154 | 125 | 1472 | 1351 | 957 |  | 324 | 344 | 7 | 261 | 65 | 274 | 19 | 38 | 195 |  | 78 | 127 | 325 | 633 | 55 | 138 | 206 | 385 | 101 | 296 | 28 | 158 | 152 | 335 | 122 |  |
| **16** | 374 | 90 | 159 |  | 113 | 150 | 76 |  |  | 925 | 184 | 158 | 1815 | 1598 | 1061 |  | 403 | 386 | 13 | 339 | 85 | 274 | 26 | 42 | 230 |  | 103 | 163 | 378 | 664 | 78 | 153 | 245 | 455 | 109 | 373 | 33 | 185 | 179 | 425 | 141 |  |
| **17** |  | 101 | 187 |  | 131 | 190 | 89 |  |  | 1146 | 220 | 184 | 2136 | 1784 | 1302 |  | 468 | 440 | 23 | 382 | 130 | 330 | 37 | 52 | 291 |  | 144 | 190 | 430 | 679 | 100 | 185 | 312 |  | 140 | 439 |  | 279 | 205 | 481 | 164 |  |
| **18** |  |  | 305 |  |  |  |  |  |  | 1307 | 256 | 202 | 2368 | 2122 | 1344 |  | 482 |  | 23 | 465 | 143 | 378 | 52 | 62 | 333 |  | 171 | 251 | 518 | 710 | 114 | 241 | 363 |  |  | 523 |  | 327 | 248 | 626 | 235 |  |
| **19** |  |  | 305 |  |  |  |  |  |  | 1551 | 286 | 234 | 2864 | 2473 | 1565 |  | 622 |  | 45 | 514 | 224 |  | 60 | 87 | 395 |  |  | 306 | 570 | 853 | 124 | 299 | 398 |  |  | 611 |  | 376 | 282 |  | 287 |  |
| **20** |  |  | 359 |  |  |  |  |  |  | 1750 |  | 265 | 3416 | 2918 | 1792 |  | 722 |  | 45 |  | 327 |  | 60 | 96 |  |  |  | 367 | 662 | 1012 | 153 | 414 | 425 |  |  | 658 |  | 413 | 328 |  | 325 |  |
| **21** |  |  | 542 |  |  |  |  |  |  | 1983 |  |  |  | 3300 | 1881 |  | 801 |  | 46 |  | 346 |  | 65 | 96 |  |  |  | 440 | 707 | 1073 |  |  | 509 |  |  | 715 |  | 452 | 356 |  | 393 |  |
| **22** |  |  | 749 |  |  |  |  |  |  | 2326 |  |  |  | 3784 | 2073 |  | 884 |  | 74 |  | 376 |  | 83 | 100 |  |  |  | 460 | 800 | 1204 |  |  | 509 |  |  | 781 |  | 502 | 378 |  |  |  |
| **23** |  |  | 1042 |  |  |  |  |  |  | 2644 |  |  |  |  | 2167 |  | 978 |  | 75 |  | 401 |  |  | 126 |  |  |  | 575 | 800 | 1340 |  |  | 613 |  |  | 882 |  | 591 | 426 |  |  |  |
|  | **AL** | **AT** | **TO** | **CN** | **VC** | **NO** | **VB** | **BI** | **AO** | **MI** | **CO** | **VA** | **BG** | **BS** | **CR** | **LO** | **PV** | **LC** | **SO** | **MN** | **MB** | **GE** | **SP** | **SV** | **BO** | **FE** | **FC** | **MO** | **PR** | **PC** | **RA** | **RE** | **RI** | **TN** | **BL** | **PD** | **RO** | **TV** | **VE** | **VR** | **VI** | Median |
| **SN** | 1 | 37 | 2 | 1 | 7 | 5 | 5 | 3 | 2 | 8 | 5 | 4 | 18 | 14 | 53 | 125 | 27 | 8 | 7 | 5 | 11 | 1 | 1 | 19 | 3 | 8 | 3 | 3 | 4 | 18 | 3 | 1 | 1 | 7 | 7 | 30 | 1 | 2 | 8 | 2 | 5 | **5** |
| **ST (SN= 5)** | 2 | 1 | 4 | 3 | 4 | 6 | 3 | 3 | 2 | 1 | 5 | 5 | 1 | 2 | 1 | 1 | 1 | 4 | 12 | 3 | 3 | 6 | 11 | 2 | 4 | 3 | 7 | 2 | 2 | 1 | 8 | 3 | 3 | 2 | 2 | 1 | 3 | 3 | 1 | 2 | 6 |  |
