# Supplementary material for: Can particulate matter be identified as the primary cause of the rapid spread of CoViD-19 in some areas of Northern Italy?
Source: Environ Sci Pollut Res Int. 2021 Feb 26;28(25):33120–32. doi: 10.1007/s11356-021-12735-x (PMC7909738; doi:10.1007/s11356-021-12735-x)
Supplement: Supplementary file 4 — (DOCX 29 kb) [file 11356_2021_12735_MOESM4_ESM.docx]

**Can particulate matter be identified as the primary cause of the rapid spread of CoViD-19 in some areas of Northern Italy?**

Maria Cristina Collivignarelli ^1,2^, Alessandro Abbà ^3^, Francesca Maria Caccamo ^1^, Giorgio Bertanza ^3^, Roberta Pedrazzani ^4^, Marco Baldi ^5^, Paola Ricciardi ^1^, Marco Carnevale Miino ^1,*^

^1^: Department of Civil Engineering and Architecture, University of Pavia, via Ferrata 3, 27100 Pavia, Italy

^2^: Interdepartmental Centre for Water Research, University of Pavia, via Ferrata 3, 27100 Pavia, Italy

^3^: Department of Civil, Environmental, Architectural Engineering and Mathematics, University of Brescia, via Branze 43, 25123 Brescia, Italy

^4^: Department of Mechanical and Industrial Engineering, University of Brescia, via Branze 38, I-25123, Brescia, Italy

^5^: Department of Chemistry, University of Pavia, viale Taramelli 10, 27100 Pavia, Italy

**^*^**: Corresponding author -> Email address: marco.carnevalemiino01@universitadipavia.it (Marco Carnevale Miino)

**Table S3** Values of parameter I_0_, a_1_ and a_2_ of the fitted curve for each city. n: number of epidemiological data used; AL: Alessandria; AO: Aosta; AT: Asti; BG: Bergamo; BI: Biella; BL: Belluno; BO: Bologna; BS: Brescia; CN: Cuneo; CO: Como; CR: Cremona; FC: Forlì and Cesena; FE: Ferrara; GE: Genoa; LC: Lecco; LO: Lodi; MB: Monza; MI: Milan; MN: Mantova; MO: Modena; NO: Novara; PC: Piacenza; PD: Padua; PR: Parma; PV: Pavia; RA: Ravenna; RE: Reggio Emilia; RI: Rimini; RO: Rovigo; SO: Sondrio; SP: La Spezia; SV: Savona; TN: Trento; TO: Turin; TV: Treviso; VA: Varese; VB: Verbania; VC: Vercelli; VE: Venice; VI: Vicenza; VR: Verona

|  | **I_0_** | | **a_1_** | | **a_2_** | | **Statistics** | | | **n** |
| --- | --- | --- | --- | --- | --- | --- | --- | --- | --- | --- |
|  | **Value** | **Standard Error** | **Value** | **Standard Error** | **Value** | **Standard Error** | **Reduced Chi-Sqr** | **R^2^** | **Adj. R^2^** | **Value** |
| **AL** | -22.7858 | 9.56625 | 26.19837 | 5.03171 | 5.85865 | 0.38588 | 75.97882 | 0.99504 | 0.99427 | 16 |
| **AO** | 1.71263 | 4.29319 | 1.8668 | 0.83855 | 3.09808 | 0.30763 | 53.16056 | 0.98344 | 0.98043 | 14 |
| **AT** | -21.7287 | 35.72876 | 55.13212 | 33.69611 | 21.54214 | 9.00583 | 11.14741 | 0.97627 | 0.97288 | 17 |
| **BG** | -203.838 | 35.39495 | 158.0004 | 15.60995 | 6.38705 | 0.19475 | 2007.194 | 0.99831 | 0.99811 | 20 |
| **BI** | -7.17961 | 11.79803 | 12.64229 | 7.63165 | 5.90517 | 1.4274 | 44.89418 | 0.96438 | 0.95726 | 13 |
| **BL** | -24.2667 | 14.23002 | 28.4825 | 10.79813 | 7.51038 | 1.33604 | 27.44821 | 0.98677 | 0.98412 | 13 |
| **BO** | -27.7766 | 6.76252 | 26.96441 | 3.52543 | 6.16487 | 0.27473 | 41.71881 | 0.99751 | 0.99715 | 17 |
| **BS** | -506.372 | 128.0566 | 335.9424 | 72.69268 | 7.38954 | 0.57685 | 13351.57 | 0.99211 | 0.99112 | 19 |
| **CN** | -7.13864 | 3.36307 | 7.11377 | 1.45886 | 3.86534 | 0.24313 | 10.34555 | 0.99634 | 0.99553 | 12 |
| **CO** | -51.1039 | 13.8124 | 41.47427 | 9.227 | 7.04331 | 0.64468 | 63.68357 | 0.99396 | 0.99296 | 15 |
| **CR** | -444.391 | 106.4128 | 388.9752 | 76.50944 | 11.78469 | 1.02075 | 4300.61 | 0.99211 | 0.99132 | 23 |
| **FC** | -4.28593 | 3.55282 | 6.98978 | 1.44325 | 4.00542 | 0.24502 | 14.35589 | 0.99591 | 0.99509 | 13 |
| **FE** | -20.5545 | 12.1958 | 24.86144 | 10.44571 | 7.24568 | 1.69718 | 3.85034 | 0.9926 | 0.99013 | 9 |
| **GE** | -93.1257 | 52.88475 | 67.80099 | 37.16547 | 7.07982 | 1.67595 | 666.514 | 0.96818 | 0.96239 | 14 |
| **LC** | -152.931 | 58.68616 | 128.1065 | 46.81082 | 8.33753 | 1.52105 | 316.3185 | 0.9884 | 0.98608 | 13 |
| **LO** | 112.6962 | 7.52882 | 6.44749 | 2.38324 | 1.67774 | 0.1642 | 34.68078 | 0.99701 | 0.99502 | 6 |
| **MB** | -64.8898 | 50.70504 | 56.87494 | 33.2301 | 6.87361 | 1.62444 | 935.1566 | 0.9596 | 0.95286 | 15 |
| **MI** | -218.149 | 53.81135 | 127.9403 | 23.79022 | 7.01722 | 0.40309 | 5133.701 | 0.99328 | 0.99258 | 22 |
| **MN** | -43.6413 | 14.93506 | 36.60401 | 7.77233 | 6.15611 | 0.44505 | 204.4159 | 0.99345 | 0.99252 | 17 |
| **MO** | -11.8609 | 7.01142 | 12.65234 | 2.13228 | 5.98176 | 0.26129 | 161.3006 | 0.99479 | 0.99427 | 23 |
| **NO** | 3.00223 | 4.49039 | 4.4393 | 1.37037 | 3.19044 | 0.25618 | 31.73091 | 0.99258 | 0.99093 | 12 |
| **PC** | -373.065 | 113.1096 | 372.6174 | 92.91388 | 15.40336 | 1.98866 | 1803.724 | 0.98904 | 0.98794 | 23 |
| **PD** | -46.9666 | 21.64301 | 73.40548 | 12.44327 | 8.99543 | 0.55457 | 455.2913 | 0.99386 | 0.99324 | 23 |
| **PR** | -173.927 | 34.89795 | 138.7576 | 24.62346 | 11.44977 | 0.87783 | 513.0932 | 0.9935 | 0.99285 | 23 |
| **PV** | -61.2791 | 16.11574 | 65.57775 | 8.37606 | 8.23669 | 0.35734 | 336.5017 | 0.99654 | 0.99619 | 23 |
| **RA** | -23.8434 | 9.92571 | 20.43347 | 6.50799 | 6.44118 | 0.83062 | 32.86302 | 0.98875 | 0.9867 | 14 |
| **RE** | 1.80809 | 10.56138 | 7.45922 | 3.03678 | 5.09053 | 0.52539 | 335.1456 | 0.97534 | 0.97243 | 20 |
| **RI** | -85.7284 | 23.86018 | 54.49339 | 13.64294 | 8.94897 | 0.81163 | 562.9489 | 0.9867 | 0.98537 | 23 |
| **RO** | -9.78107 | 8.42107 | 9.85551 | 6.8669 | 10.6936 | 3.82713 | 6.93584 | 0.94781 | 0.93978 | 16 |
| **SO** | -78.4543 | 136.418 | 79.32888 | 130.0514 | 13.37329 | 15.60024 | 43.82122 | 0.94583 | 0.92778 | 9 |
| **SP** | -43.1566 | 28.00249 | 39.82091 | 24.72758 | 11.38296 | 4.14596 | 21.93298 | 0.97424 | 0.96908 | 13 |
| **SV** | -92.6215 | 101.0521 | 103.7372 | 95.84453 | 16.44174 | 10.60172 | 45.43596 | 0.96949 | 0.96271 | 12 |
| **TN** | -113.287 | 79.63028 | 79.49209 | 55.11129 | 6.92341 | 2.04215 | 1637.294 | 0.94971 | 0.94057 | 14 |
| **TO** | 13.57864 | 9.65289 | 1.79883 | 0.55445 | 3.32021 | 0.16506 | 742.3103 | 0.99129 | 0.99033 | 21 |
| **TV** | -24.0203 | 17.47266 | 33.73167 | 8.57921 | 7.54898 | 0.62971 | 432.7009 | 0.98722 | 0.98588 | 22 |
| **VA** | -37.7254 | 12.556 | 29.99095 | 7.77165 | 7.24617 | 0.72346 | 83.98631 | 0.99022 | 0.98882 | 17 |
| **VB** | 1.11155 | 2.77126 | 3.07929 | 1.04967 | 4.12985 | 0.40279 | 10.71401 | 0.98797 | 0.98578 | 14 |
| **VC** | -2.82532 | 10.71924 | 6.05804 | 4.54736 | 4.1137 | 0.93551 | 120.7621 | 0.94793 | 0.93752 | 13 |
| **VE** | -55.9802 | 11.48888 | 51.83659 | 7.43029 | 9.7656 | 0.55834 | 79.68585 | 0.99586 | 0.99543 | 22 |
| **VI** | -23.0628 | 8.38996 | 22.98364 | 4.06789 | 5.49816 | 0.31728 | 71.52502 | 0.99584 | 0.9952 | 16 |
| **VR** | -14.7726 | 7.51136 | 14.06908 | 2.28671 | 4.71441 | 0.19884 | 141.3756 | 0.99641 | 0.99593 | 18 |
